# Inferring Biological Mechanisms by Data-Based Mathematical Modelling: Compartment-Specific Gene Activation during Sporulation in *Bacillus subtilis* as a Test Case

**DOI:** 10.1155/2011/124062

**Published:** 2012-01-23

**Authors:** Dagmar Iber

**Affiliations:** Department for Biosystems Science and Engineering, Switzerland and Swiss Institute of Bioinformatics (SIB), ETH Zurich, Mattenstraße 26, Basel 4058, Switzerland

## Abstract

Biological functionality arises from the complex interactions of simple components. Emerging behaviour is difficult to recognize with verbal models alone, and mathematical approaches are important. Even few interacting components can give rise to a wide range of different responses, that is, sustained, transient, oscillatory, switch-like responses, depending on the values of the model parameters. A quantitative comparison of model predictions and experiments is therefore important to distinguish between competing hypotheses and to judge whether a certain regulatory behaviour is at all possible and plausible given the observed type and strengths of interactions and the speed of reactions. Here I will review a detailed model for the transcription factor *σ* ^*F*^, a regulator of cell differentiation during sporulation in *Bacillus subtilis*. I will focus in particular on the type of conclusions that can be drawn from detailed, carefully validated models of biological signaling networks. For most systems, such detailed experimental information is currently not available, but accumulating biochemical data through technical advances are likely to enable the detailed modelling of an increasing number of pathways. A major challenge will be the linking of such detailed models and their integration into a multiscale framework to enable their analysis in a larger biological context.

## 1. Introduction

The success of modern physics came about by a fruitful combination of theory and experiment. Models in physics succeed, in general, in predicting experimental results quantitatively. Where applicable, concepts from physics and chemistry have also greatly helped to understand biological mechanisms, the generation of ATP by coupling phosphorylation to an electrochemical concentration gradient [1], the emergence of action potentials based on changes in membrane conductivity [2], and the kinetics of enzymatic reactions [3], among many others. However, in most cases it is the regulatory structure that emerges from a complex network of protein and gene interactions that determines biological functionalities and appearances. Jacob and Monod were the first to recognize the regulatory logic of a gene regulatory network [4], a network that has since attracted numerous computational studies and has led to the discovery of many important concepts in molecular biology [5]. Further theoretical studies have established the basic requirements for a range of qualitative properties of the regulatory system, that is, its ability to show transient, sustained, or oscillatory responses, or to be sensitive or robust to molecular noise [6, 7]. While the basic requirements are now mostly understood, their functioning in the complex setting of a cell has remained hazy.

**Figure 1:**
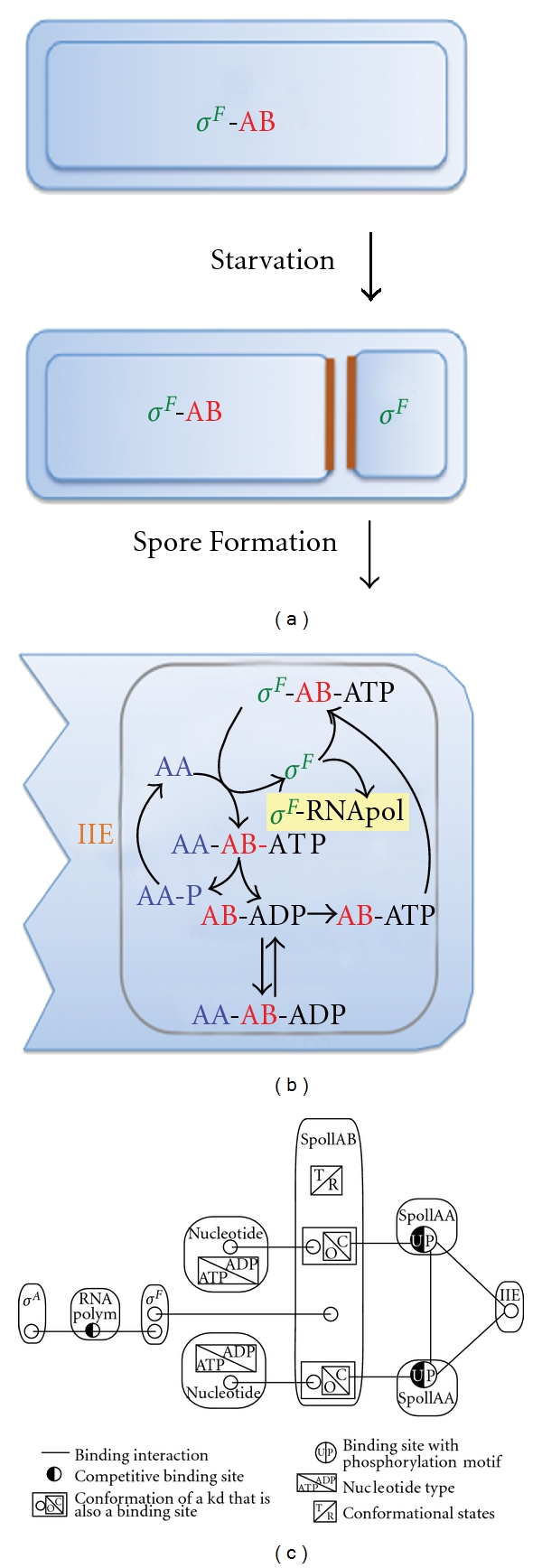
The regulation of the transcription factors *σ* ^*F*^ during sporulation in *Bacillus subtilis*. (a) Differential cell fate during sporulation in *B. subtilis*: in response to starvation, *B. subtilis* divides asymmetrically and *σ* ^*F*^ becomes active in the smaller compartment. (b) The regulatory networks that controls *σ* ^*F*^. (c) A contact map of the regulatory network. All possible interactions between regulatory components are indicated. Boxes represent complexes. Circles represent binding sites, and connecting lines indicate binding interactions. Circles that are both black and white demonstrate binding sites for competing ligands. (a) and (b) have been reproduced from [47].

Careful experiments in biochemistry, genetics, and molecular biology have defined the key signaling pathways and networks that regulate biological responses and have provided information about the mode of interaction as well as about the kinetics of catalysed reactions. The discovered pathways and core networks are typically limited to less than ten components and can therefore be captured by simple cartoons as depicted in Figure 1 for the network that regulates the transcription factor *σ* ^*F*^ during sporulation in *Bacillus subtilis*. In spite of much detailed information, mechanisms that emerge from the dynamic interaction of components, it can be difficult to derive by verbal reasoning alone, in particular when the regulatory mechanism is sensitive and thus requires only small changes in the regulatory parameters.

Mathematical models can be employed to integrate the available information into a formal framework that is amenable to the complexity of biological reality. The level of detail of a model is dictated by the question under investigation and by the type of data that is available to test the model with. Many theoretical studies make use of simplified phenomenological models since they can be analysed and explored more comprehensively. Since the parameters in phenomenological models typically do not directly relate to measurable quantities, it is, however, difficult to assign realistic values to the parameters in these models. The extent of cooperativity in binding interactions and the resulting nonlinearity are often overestimated and a separation of time scales is assumed when this is not warranted. To understand the mechanistic details of biological regulation to an extent that the system can be externally controlled and manipulated typically requires a more quantitative understanding [8]. A quantitative comparison of model predictions and experiments may also be necessary to distinguish between competing hypotheses and to judge whether a certain regulatory behaviour is at all possible and plausible given the observed type and strengths of interactions and the speed of reactions. We will illustrate the use of both detailed, quantitative as well as phenomenological, qualitative models by discussing the regulatory control of *σ* ^*F*^ during sporulation in *Bacillus subtilis*.

## 2. Cell Differentiation in *Bacillus subtilis*

Sporulation in *B. subtilis* is one of the best understood examples for cell differentiation and development and has provided a paradigm for asymmetric cell division and differential cell fate decisions in genetically identical sister cells [9]. In response to starvation, *B. subtilis* can initiate a cellular program that leads to asymmetric cell division and to the subsequent differentiation of the smaller compartment (prespore or forespore) into an endospore that can withstand and survive particularly harsh conditions (Figure 1(a)). The larger sister cell develops into an altruistic mother cell that supports the development of the prespore. The different fates of the two compartments are sealed when the transcription factor *σ* ^*F*^ is activated in the smaller but not in the larger compartment [10–12]. The network that controls *σ* ^*F*^ activity is simple (Figure 1(b)) and has been known for a long time [12]. Yet how compartment-specific activation of *σ* ^*F*^ is achieved has long remained elusive.

The transcription factor *σ* ^*F*^ is controlled by a 3-component network which comprises the kinase SpoIIAB (AB), the phosphatase SpoIIE (IIE), and the common substrate SpoIIAA (AA) [13–17]. AB sequesters *σ* ^*F*^ in an inactive complex [16, 18], and binding of AA leads to the rapid release of the transcription factor [19–21]. AB then uses the ATP in its nucleotide-binding pocket to phosphorylate AA [16, 22, 23]. Phosphorylation causes a rapid dissociation of AA, and AA needs to be dephosphorylated by the phosphatase IIE [13–15] before it can bind to AB and release *σ* ^*F*^. To rebind *σ* ^*F*^ with high affinity AB needs to exchange ADP for ATP in its nucleotide-binding pocket [23, 24]. Binding of AA to ADP-bound results in the sequestration of AB in a stable, long-lived complex because AA cannot be phosphorylated [23, 25, 26].

Before septation most AA is phosphorylated [23, 27]. Upon septation, the phosphatase IIE accumulates on the septum between mother cell and prespore [13, 28], and unphosphorylated AA emerges [23, 27]. It has remained controversial whether IIE preferentially accumulates on the side of the septum that faces the prespore compartment [28–34]. However, because of the difference in size, the activity of the phosphatase increases in the smaller compartment also when IIE accumulates homogenously on both sides of the septum [13, 28]. Since such increase would, however, be small (about 4-fold), based on verbal reasoning, it remained unclear whether the resulting higher concentration of unphosphorylated AA would be sufficient to trigger *σ* ^*F*^ release in the smaller compartment. A number of alternative mechanisms have been considered.

A transient imbalance between mother cell and prespore was proposed to arise from a transient unequal distribution of the chromosomes. The chromosomal part that encodes the genes for AA, AB, and *σ* ^*F*^ (spoIIA operon) remains in the mother cell for the first 10–15 minutes after septation [35]. As a result AA, AB, and *σ* ^*F*^ can initially not be expressed in the prespore, and it has been suggested that this may lead to a relative increase in the concentration of the phosphatase relative to these components [36]. The expression of spoIIA may also be repressed in the prespore compartment by emerging unphosphorylated AA, a potent inhibitor of Spo0A activation [37].

Further contributions have been proposed to enhance any small asymmetries. Firstly, a starvation-induced drop in the ATP concentration has been suggested to hamper the ADP-ATP exchange at the catalytic side of AB [25]. Since ADP-bound AB needs to exchange ADP for ATP to avoid sequestration in an inactive complex with AA, it has been argued that a starvation-induced drop in the ATP concentration might favour *σ* ^*F*^ release [25]. The physiological range within which the ATP concentration changes is, however, small (0.8–3 mM) [38–40]. Additionally, the protease ClpCP has been found to target unbound AB for degradation, and this has been suggested to lower the concentration of AB that is available to rebind *σ* ^*F*^ after AA-induced dissociation of the complex [41, 42]. Recently, it has been suggested that ClpCP acts preferentially in the forespore and that this may bias *σ* ^*F*^ release to this compartment [43]. However, the AB half-life of about 28 minutes is much longer than the time within which active *σ* ^*F*^ first emerges in the cell (10 minutes).

In spite of many elegant experiments, it remained impossible to judge whether the aforementioned contributions would be sufficient to enable septation-dependent and compartment-specific *σ* ^*F*^ release, or whether further important regulatory interactions had been overlooked. Moreover, the distinct contributions of the many effects to the physiological regulation of *σ* ^*F*^ remained difficult to evaluate with verbal models. Mathematical methods in combination with experiments were thus the method of choice to address the problem.

## 3. The Development and Validation of a Mathematical Model

The regulatory system was particularly amenable to a quantitative analysis because all network components could be purified and the network could therefore be reconstituted in the test tube [44, 45]. This permitted us to develop a comprehensive differential equation model that would include all states and reactions of the test tube network [46, 47]. As we intended to create a quantitative, predictive model, it was important to move away from phenomenological descriptions to a detailed, mechanistic model that considered all binding reactions and conformational changes explicitly.  Figure 1(c) shows a contact map of all possible regulatory interactions in the network. Accordingly, all parameter values corresponded to a physical entity and could be determined from experimental *in vitro* data. The detailed model for the small regulatory network with only four components (plus the RNA polymerase and the housekeeping transcription factor *σ* ^*A*^ for the cellular model) eventually comprised more than 150 reactions that gave rise to a set of about 50 differential equations. The reaction kinetics depended on about 30 independent parameter values that we measured in experiments. While we took great care to validate the model with experimental data, there always remain concerns with regard to the estimated parameter values. Does the optimized parameter set represent biological reality or are conclusions misguided by errors in the data and limitations in the parameter estimation? Parameter estimation procedures for such large systems are prone to be trapped in a local parameter optimum. To address such concerns, we have recently conducted a wider parameter screen where we searched within a larger parameter space for parameter combinations that would capture the *in vitro* data (Iber, unpublished results). We noticed that about 20–30% of the parameter sets that fitted the *in vitro* behaviour reasonably well captured also the *in vivo* behaviour. Only when we required a very accurate fit to the experimental data did we obtain a 100% success rate in our predictions of the *in vivo* behaviour. This stresses the importance of high quality, quantitative data to extract meaningful insight from a model.

## 4. The Predictive Power of Quantitative Models

**Figure 2:**
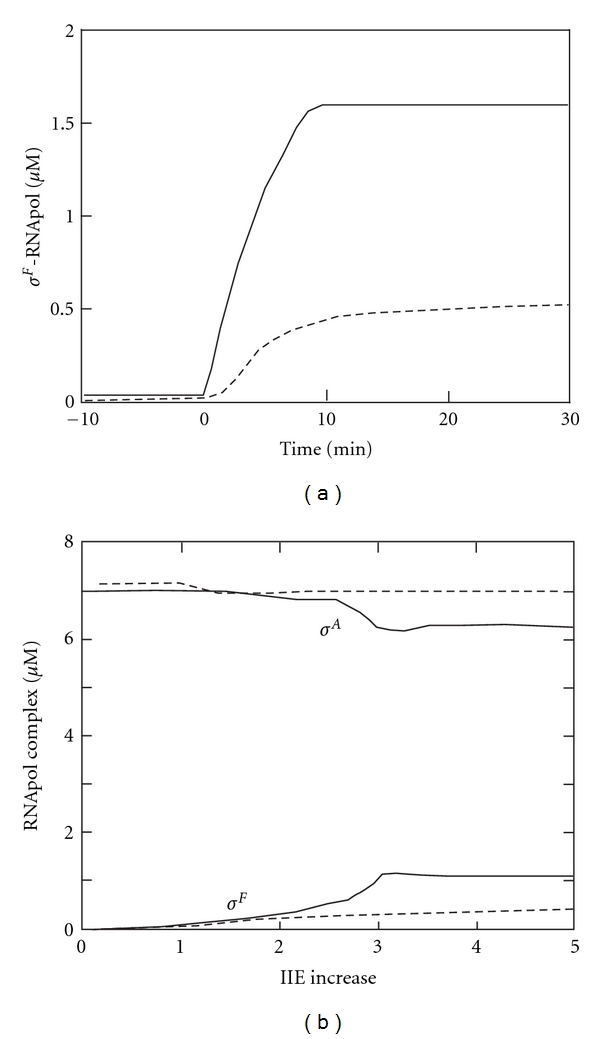
The regulation of the transcription factors *σ* ^*F*^ during sporulation in *Bacillus subtilis*. (a) The solid lines show the concentration of RNA polymerase-*σ* ^*F*^ holoenzyme before and after septation (time 0). Septation was modelled as a 4-fold increase in the concentration of the phosphatase IIE. The dashed lines show the model prediction if AB is not allosteric with both AA and *σ* ^*F*^ binding with high affinity. (b) Predicted concentration of *σ* ^*F*^ (lower curves) or *σ* ^*A*^ (upper curves) RNA polymerase holoenzyme formed 90 min after septation, as a function of an increase in IIE (including IIE complexed with AA-P) relative to the other sporulation proteins after 120 min of protein expression, assuming that AB is allosteric (continuous lines) or not allosteric with both AA and *σ* ^*F*^ binding with high affinity (dashed lines). (a) and (b) have been reproduced from [47].

Based on the fully parameterized and validated model, we predicted that the difference in cell size would be sufficient to determine cell fate [47]. A 2.5-fold increase in the phosphatase concentration was sufficient to trigger the appearance of micromolar concentrations of RNA polymerase-*σ* ^*F*^ holoenzyme in the model (Figure 2). The model was not only validated with *in vitro* data but also succeeded in predicting the phenotypes of all mutants for which quantitative data was available [46, 47]. This was important because it enabled us to show that also those experiments that had led to alternative proposals could be reproduced with our model, and that the other proposed mechanisms would not contribute significantly to the control of *σ* ^*F*^ release under physiological conditions (i.e., for physiologically realistic parameter values). Thus neither the proposed temporal imbalance in gene expression [36] nor AB degradation [41, 42] is relevant on the time scale on which *σ* ^*F*^ becomes active [46, 47]. Equally the same response is attained for the entire range of physiological ATP concentrations. A lower starvation-induced ATP concentration, therefore, does not play a role in *σ* ^*F*^ activation [25]. Once we had shown that the regulatory interactions in Figures 1(b) and 1(c) were sufficient to explain septation-dependent *σ* ^*F*^ activation, the model could be used to explain how this extraordinary high sensitivity to changes in the phosphatase concentration could be achieved. We realised that a combination of allosteric effects and enzyme saturation enables this high sensitivity with a small 3-component network.

### 4.1. Allosteric Effects

**Figure 3:**
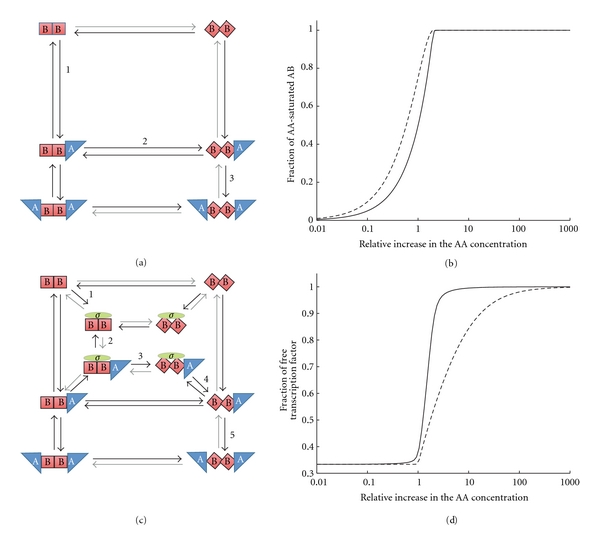
Allosteric interactions enhance sensitivity. (a) The interactions between AB (red) and AA (blue) constitute an allosteric binding mechanism. At low AA concentrations little AA can bind to AB. As the AA concentrations increase, more AB-AA complexes form (1). Binding of AA biases the AB dimer into a different conformation (2) that binds AA with high affinity, and a second AA binds to the AB dimer (3). (b) The allosteric binding mechanism enhances the sensitivity to changes in the AA concentration. The solid line shows the fraction of AA-saturated AB dependent on the AA concentration when we use the physiological affinities. The dashed line shows the fraction of AA-saturated AB if AB had only one conformation and interacted with AA with high affinity. (c) The interactions between AB (red), AA (blue), and *σ* ^*F*^ (green) lead to a sophisticated allosteric control mechanism. At low AA concentrations *σ* ^*F*^ can bind to AB (1). As the AA concentrations increase, AB-*σ* ^*F*^-AA complexes form (2). Binding of AA biases the AB dimer into a different conformation (3) that binds *σ* ^*F*^ with low and AA with high affinity. *σ* ^*F*^ thus rapidly unbinds (4) and a second AA binds to the AB dimer (5). (d) The sophisticated allosteric binding mechanism enhances the sensitivity to changes in the AA concentration. The solid line shows the fraction of unbound *σ* ^*F*^ dependent on the AA concentration when we use the physiological affinities. The dashed line shows the fraction of unbound *σ* ^*F*^ if AB had only one conformation and interacted both with *σ* ^*F*^ and AA with high affinity.

Allosteric effects (and the resulting cooperativity) have long been recognised to enable increased sensitivity [49]. Allosteric enzymes harbour several ligand-binding sites and the different conformations that the protein can assume bind ligand with different affinities *K*_*i*_. Ligand binding alters the conformational equilibrium and therefore either increases or reduces the affinity of binding, resulting in either positive or negative cooperativity, respectively [3]. This is illustrated in Figure 3(a) by example of the AB protein which has two binding sites for AA. Unbound AB is mainly in a conformation (denoted by squares in Figure 3(a)) that binds AA with low affinity, that is, the AB-AA off-rate is large. Binding of the first AA alters the conformational equilibrium in that a higher fraction of AB now attains a conformation (denoted by rhombs in Figure 3(a)) that binds AA with high affinity, that is, low AB-AA off-rate (grey arrows in Figure 3(a)). This favours the binding of a second AA. As a result little AB is bound at low AA concentrations. Once a critical AA concentration is reached to enable binding of the first AA, binding of the second AA is facilitated by the conformational change. As a result the binding profile changes from mainly unbound to mainly bound over a smaller AA concentration range (Figure 3(b), solid line) compared to a mechanism where the binding of the two ligands is independent (Figure 3(b), broken line). In phenomenological models, Hill functions of the form *y* = [*X*]^*n*^/(*K* ^*n*^ + [*X*]^*n*^) are typically used to model the fraction of allosteric protein (enzyme) binding sites *y* that are bound by ligand *X*. The Hill constant *K* denotes the ligand concentration *X* at which half of the allosteric binding sites are bound by ligand, while the Hill coefficient *n* determines the sensitivity to the ligand concentration. Often this sensitivity is overestimated by using large *n*. However, *n* cannot be larger than the total number of ligand-binding sites, and typically is much smaller; a more detailed discussion can be found in standard text books in mathematical biology and protein science [3, 50]. Figure 3(b) illustrates that even though the different sensitivities of the allosteric and independent binding mechanism can easily be noted, the difference is not particularly large for physiological parameters.

We discovered a sophisticated modification of the standard allosteric mechanism that can lead to a particularly sensitive switch-like behaviour (Figures 3(c) and 3(d)). AB is an unusual allosteric protein in that the dimer binds two different ligands, AA and *σ* ^*F*^ (Figure 3(c)). There is only one binding side for *σ* ^*F*^, which binds across the interface of the dimer, while there are two binding sites for AA [51]. Only AA induces a conformational change in the AB dimer, but this conformational change also alters the AB-*σ* ^*F*^ affinity. Importantly the AA-induced conformational change enhances the AA-AB affinity but lowers the AB- *σ* ^*F*^ affinity. Accumulation of AA, therefore, biases AB to a conformation that binds *σ* ^*F*^ with low affinity and thus facilitates its release. This change in AB-*σ* ^*F*^ affinity not only enhances the sensitivity of *σ* ^*F*^ to a change in the AA concentration (Figure 3, compare solid (change in AB-*σ* ^*F*^ affinity) and broken (only high AB-*σ* ^*F*^ affinity) lines in panel (d) but also enhances the sensitivity of the AB-*σ* ^*F*^ complex towards changes in the AA concentration (Figure 3(d), solid line) beyond that of the AB-AA interaction (Figure 3(b), solid line). This particular allosteric mechanism thus greatly enhances the sensitivity of the AB-*σ* ^*F*^ complex to AA (Figure 3, compare panels (b) and (d) to a switch-like response and explains the rapid, AA-induced dissociation of AB-*σ* ^*F*^ that is observed in experiments [19–21].

### 4.2. Bistability and Hysteresis

A switching behaviour during cell differentiation has also been accounted to bistability [52]. Bistability arises when a dynamic system has more than one steady state and a change in a so-called bifurcation parameter that either renders the current steady state unstable or removes it altogether [53]. The system then jumps from the initial steady-state branch to a new branch as the bifurcation parameter passes the bifurcation point. Since the change happens at a single point the systems is highly sensitive to changes in the bifurcation parameter close to this bifurcation point. In case of hysteresis, the system does not switch back at the same bifurcation point. A larger change in the bifurcation parameter is required to bring the system back to its initial state, and it is possible to generate systems that do not switch back within the physiological parameter range. Given the combination of high sensitivity and robustness, bistability and hysteresis provide an attractive mechanism to explain cell differentiation [54, 55].

Bistability has also been argued to be important for heterogeneity in cellular decision making. Since two stable steady states exist in a bistable system, molecular noise can result in a heterogenous fate within a cellular population. As a result not all members of a population take the same differentiation path and changes in external conditions can be withstood more easily on the population level (bet-hedging strategy) [56]. A bimodal response has indeed been observed also in starving *B. subtilis* cultures, and it is thought that the bimodal activation levels of the Master transcription factor Spo0A are the result of both negative regulatory effects of phosphatases such as Spo0E and RapA and autostimulatory Spo0A loops [57–59]. Activation of Spo0A affects the transcription of more than 100 genes, among these the *spoIIA* operon and *spoIIE* [60].

The analysis of greatly simplified, phenomenological models suggests that also the *σ* ^*F*^ signaling network exhibits bistability and hysteresis [61, 62]. A bimodal activation profile of *σ* ^*F*^ would, however, be detrimental because asymmetric septation and compartment-specific activation of *σ* ^*F*^ happen only about 2 hours after Spo0A has first been activated. At this time, the cell is already fully committed to the sporulation program and failed activation of *σ* ^*F*^ will result in cell death. Bistability is a steady-state behaviour, but more than the steady-state behaviour the rapid kinetics appear to be particularly important in the regulation of *σ* ^*F*^. *σ* ^*F*^ is activated within 10 minutes of septum formation and mainly serves to initiate compartment-specific programs of gene expression both in the prespore and in the mother cell [63]. Spores have typically already been engulfed within another 90 min [64]. The entire sporulation process takes about 6–8 h [65].

The current focus of mathematical models on the steady-state behaviour mainly stems from the lack of good analytical techniques to investigate the dynamical behaviour. Given the likely importance of pre-steady-state dynamics in many cellular signalling systems, a greater focus on the dynamical aspects will be important to understand the details of biological regulation.

### 4.3. Ultrasensitivity

Almost 30 years ago, Goldbeter and Koshland coined the concept of an ultrasensitive response [66]. In an ultrasensitive system, minute changes in enzyme activity can lead to the full activation or deactivation of a downstream system. While similar in effect, the mechanisms of bistable and ultrasensitive responses are fundamentally different. Unlike in bistable systems where the system jumps between two steady states, in an ultrasensitive system the steady state itself changes its value rapidly as the enzyme activities are altered. Moreover, ultrasensitive systems cannot exhibit hysteresis and are therefore not robust to a subsequent removal of signal. Robustness to a subsequent removal of signal may, however, not be particularly important during spore formation since once the septum is formed the proteins are confined to their compartments. Rather robustness to noise in gene expression can be expected to be crucial and we will discuss mechanisms to address this aspect in a later part of this paper.

**Figure 4:**
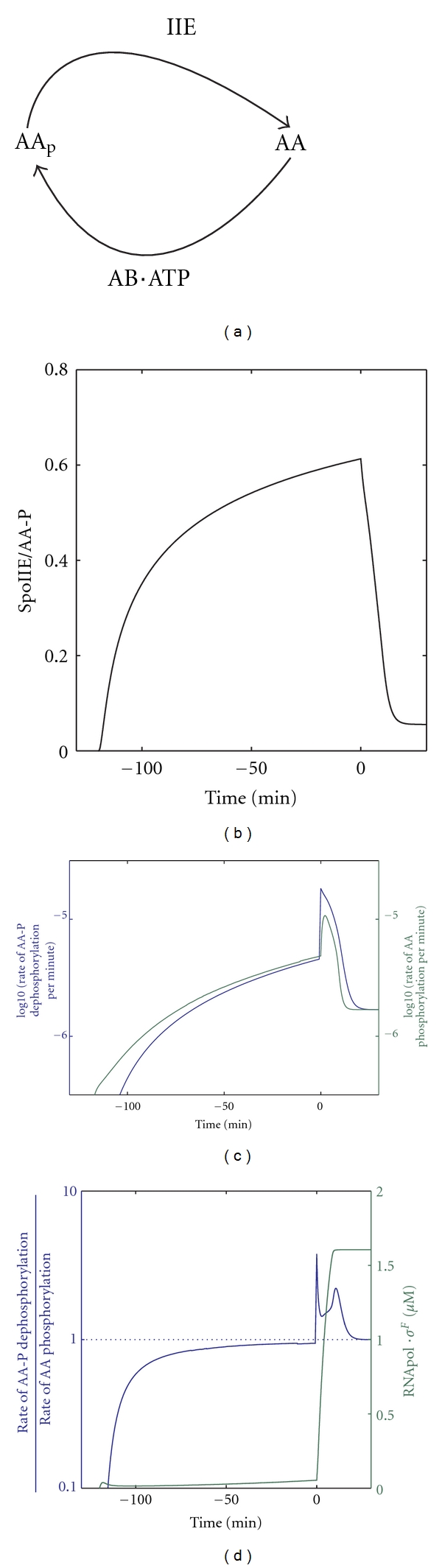
The *σ* ^*F*^ response as an ultrasensitive response. (a) The phosphorylation and dephosphorylation reactions of AA by AB and IIE, respectively, enable an ultrasensitive response. (b) The fraction of IIE that is bound in IIE/AA-P complexes over time. (c) The rate of AA-P dephosphorylation (blue line) is lower than the rate of AA phosphorylation (green line) until septation (time *t* = 0). (d) The relative rate of AA-P dephosphorylation and AA phosphorylation (blue line) is close to but below 1 until septation (*t* = 0). The stronger increase in the rate of AA-P dephosphorylation then leads to the accumulation of free *σ* ^*F*^ and binding to the RNA holoenzyme (green line). Septation (a 4-fold increase in the IIE concentration) takes place at time *t* = 0 in all panels.

As an example for an ultrasensitive response, Goldbeter and Koshland considered two enzymes with opposing activity (e.g., kinase/phosphatase, methylase/demethylase, etc.) that act on a common substrate *X* that exists in two states, say *X* and *X*_*p*_ (Figure 4(a)). In the Goldbeter-Koshland model, the kinetics of the common substrate are described by Michaels-Menten-type kinetics, that is,

(1) $$\begin{array}{c} \frac{dX}{dt}=\nu_{1}E_{1}\frac{X_{p}}{X_{p}+K_{1}}-\nu_{2}E_{2}\frac{X}{X+K_{2}}. \end{array}$$

A sensitive, switch-like response can be obtained when both enzymes (phosphatase *E*_1_, kinase *E*_2_) are saturated (i.e., their Michaelis-Menten constants are low relative to the substrate concentrations (*K*_1_/*X*_*p*_ ≪ 1, *K*_2_/*X* ≪ 1) as is achieved by a high affinity of binding and a low maximal catalytic rate (*ν*_1_, *ν*_2_)).

Suppose the common substrate, *X*, is mostly phosphorylated (i.e., *v*_max⁡_, the maximal rate times the enzyme concentration (*v*_max⁡_ = *ν*_*i*_ × *E*_*i*_), is higher for the kinase (*v*_max⁡2_ > *v*_max⁡1_)). If both enzymes are saturated with substrate, then increasing the activity of the enzyme with the lower *v*_max⁡_ (here the phosphatase) will lead to an increased substrate concentration (unphosphorylated protein in this case) for the enzyme with the higher *v*_max⁡_ (here the kinase). However, since the kinase is already saturated with substrate, this will not enhance the rate at which proteins are phosphorylated. If the *v*_max⁡_ of the phosphatase is sufficiently increased so that its *v*_max⁡_ is now higher than that of the kinase, then the proteins will switch from being mainly phosphorylated to being mainly unphosphorylated. Even a small increase in the phosphatase concentration can be sufficient to trigger such a switch if the *v*_max⁡_ of the two enzymes are similar to start with. This then results in the observed ultrasensitivity to small changes in enzyme activity.

The model predicted (and experiments confirmed) that the maximal turn-over rate of SpoIIE is very low [47]. As a result, about 60% of the phosphatase is predicted to be bound by its substrate at the time of septation (time *t* = 0, Figure 4(b)) even though the affinity of binding is low (~10 micromolar [47]). Accumulation of the phosphatase on the septum then leads to the simultaneous accumulation of substrate and thus to a strongly increased rate of AA emergence (Figure 4(c), blue line). Unlike in the Goldbeter-Koshland model, the kinase AB is not at all saturated with its substrate AA, and the emerging AA therefore results also in an increase in kinase activity (Figure 4(c), green line). However, the increase in kinase activity is smaller than the increase in phosphatase activity and since the two activities were rather similar before septation the change is sufficient for the phosphatase to suddenly dominate (Figure 4(d), blue line) and free *σ* ^*F*^ emerges that binds to the RNA polymerase holoenzyme (Figure 4(d), green line).

**Figure 5:**
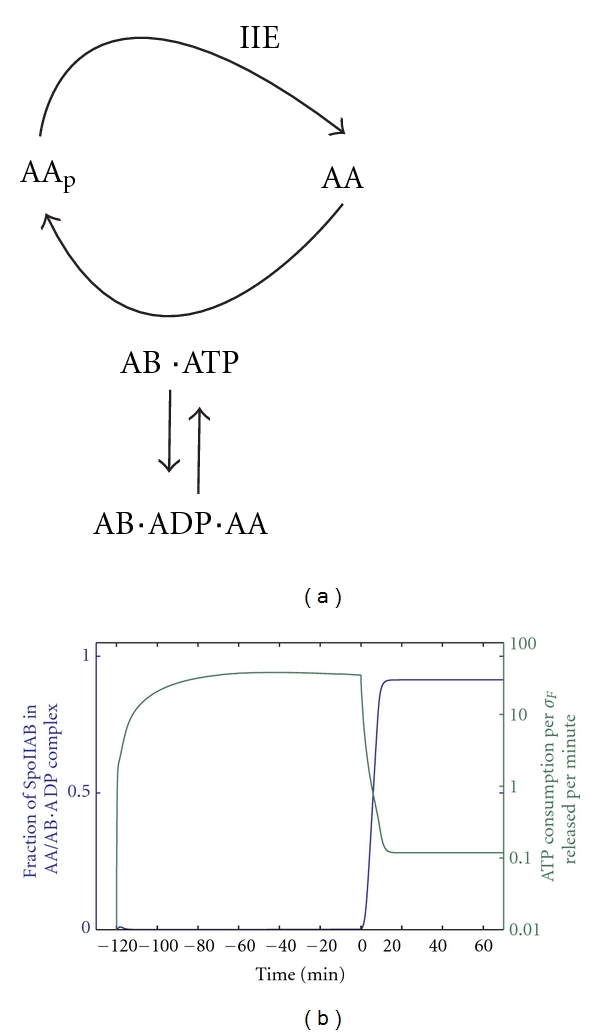
Economic efficiency. (a) Binding of AA to ADP-bound AB reduces the amount of active kinase and thus slows down the cycles of phosphorylation and dephosphorylation of AA. (b) The amount of ATP per *σ* ^*F*^ released per minute drops sharply as the fraction of AB that is sequestered in inactive AB-ADP-AA complexes sharply increases at the time of septation (time *t* = 0).

The activity of the AB kinase is limited also by sequestration of the ADP-bound form in a complex with AA (Figure 5). Activation of the transcription factor is possible also without such a sequestration step if the phosphatase activity can be raised to higher activities. This has been realised in a network that is based on homologous proteins and that achieves activation of the transcription factor *σ* ^*B*^ without forming much complex between the ADP-bound kinase and its substrate (Iber, unpublished results).

### 4.4. Economic Efficiency

Formation of the inactive complex between the ADP-bound kinase and its substrate serves a second important function, conservation of energy. Sporulation is a response to starvation, and it is therefore expected that the bacterium will limit its energy expenditure. However, activation of *σ* ^*F*^ during sporulation requires the AA-dependent release of the transcription factor *σ* ^*F*^ which results in AA phosphorylation and ATP expenditure. Importantly AA binds ADP-bound AB with high affinity, and binding of AA to AB-ADP prevents the exchange of ADP and ATP in the nucleotide-binding pocket. Since the ADP form of AB cannot phosphorylate AA, AA-AB-ADP complexes are rather stable and can act as a sink that sequesters AB and prevents it from rebinding *σ* ^*F*^ (Figure 5(a)). Cycles of ATP-consuming AA-dependent *σ* ^*F*^ release and rebinding are, therefore, avoided once AA-AB-ADP complexes form. The model indeed predicts that the amount of ATP that is required to keep one *σ* ^*F*^ released drops some hundred folds upon septation (Figure 5(b), green line) as complexes between ADP-bound AB and AA emerge (Figure 5(b), blue line) [23, 25, 26]. In the homologous network that controls *σ* ^*B*^ activity, such complexes are barely formed and almost a 100-fold more ATP is necessary to keep the transcription factor active (Iber, unpublished results). The advantage of the added energy expenditure is that the response can be reversed faster when conditions change.

### 4.5. The Evolution of Operons

**Figure 6:**
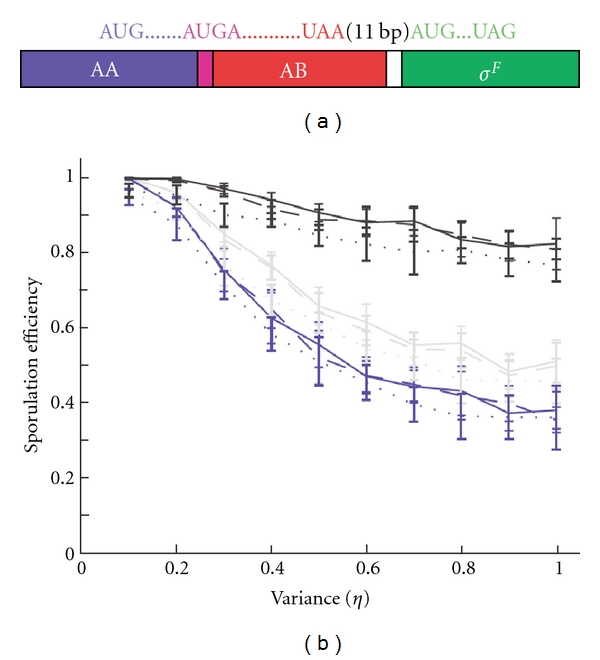
Operons and molecular noise. (a) The spoIIA operon comprises the genes for SpoIIAA, SpoIIAB, and *σ* ^*F*^. The genes for SpoIIAA and SpoIIAB overlap; the genes for SpoIIAB and *σ* ^*F*^ are separated by 11 bp. The impact of stochastic variation in gene expression on sporulation efficiency. (b) The fraction of successful sporulation events depend on the variance in gene expression if expression of the spoIIA genes is either coupled (black lines), the expression of SpoIIAB and *σ* ^*F*^ is coupled (grey lines), or the expression of SpoIIAA and *σ* ^*F*^ is coupled (blue lines). The broken lines show the effect of an additional independent normal variation in the rate of *σ* ^*F*^ expression from the coupled rates. (a) and (b) have been reproduced from [48].

Protein expression is important both in the regulation of *σ* ^*F*^ and *σ* ^*B*^. Intriguingly, both in the *σ* ^*F*^ and in the *σ* ^*B*^ network the transcription of the genes for the kinase, its substrate, and the transcription factor (but not the gene for the phosphatase) is linked by organization into an operon (Figure 6(a)). The formation and maintenance of operons has intrigued evolutionary biologists ever since their first discovery, and many theories have been put forward. Jacob and Monod proposed that the benefits of cotranscription drive operon formation [4]. Other models that focus on genetic rather than functional aspects have since been proposed to explain the selective advantage of operons, that is, the natal model, the Fisher model, and the selfish-operon model. According to the natal model, gene clustering is the consequence of gene duplication. However, since many operons comprise genes that belong to very distant families and the majority of paralogues do not cluster, this model is insufficient to explain the existence of operons [67, 68]. Similarly the selfish-operon model, which proposes that the organization of genes into operons facilitates the horizontal transfer of functionally related genes [67], does not agree with the observed gene cluster pattern [69, 70]. The Fisher model, applied to prokaryotes, proposes that clustering of genes reduces the likelihood of coadapted genes to become separated by recombination. However, since recombination is as likely to generate as to destroy clusters, this does not explain how operons can emerge.

Co-transcription provides a number of potential selective advantages. When the genes of a protein complex are encoded by an operon, co-transcription enables co-translational folding [68], it limits the half-life of toxic monomers [69], and its reduces stochastic differences in gene expression [71]. Operons that do not code for interacting proteins may be advantageous because proteins act in a cascade where they are required in defined ratios as is the case for metabolic operons [70, 72]. Evidence in favour of any of the proposed driving forces has mainly been obtained from comparative genomics.

We used our quantitative model to study the benefit of such an operon organization and noticed that in the presence of molecular noise this genetic linkage significantly increases the survival probability [48]. We compared the fraction of simulation runs with successful *σ* ^*F*^ activation upon septation (increase in IIE concentration) when the expression of AB, AA, and *σ* ^*F*^ was either correlated (Figure 6(b), black curve) or one of the three proteins was expressed independently. Since in experiments those independently expressed proteins were expressed with the same promotor, the expression rates were all drawn from a Gaussian distribution with the same mean and variance. The model predicted that at a physiological level of variance in protein expression (i.e., *η* ~ 0.6 [73]), independent expression of AB (while AA and *σ* ^*F*^ expression remained coupled) would lower the sporulation efficiency to 40–80% (Figure 6(b), blue curve), even if the same promotor (same mean and variance) was used. Such a lower sporulation efficiency has indeed been observed when the gene for AB was moved out of the operon and was expressed independently with the same promotor [74]. The model further predicted that the separate expression of AA (while AB and *σ* ^*F*^ expression remained coupled) would have a similar, yet milder effect (Figure 6(b), grey curve) as indeed observed in experiments. Independent expression of *σ* ^*F*^ is not as detrimental. This may be important since the 11 basepair distance between the AB and the *σ* ^*F*^ genes is likely to introduce additional noise in the translation of the mRNA into proteins. Here it is interesting to note that unlike the genes for AB and *σ* ^*F*^ the genes for RsbW and *σ* ^*B*^ overlap and the model for the *σ* ^*B*^ network predicts that the relative concentration of the transcriptional factor *σ* ^*B*^ and the other components is more important than in the *σ* ^*F*^ network (Iber, unpublished results).

Taken together, it appears that at least in the models for the *σ* ^*F*^ and *σ* ^*B*^ networks operon formation strongly increases fitness (survival) by reducing the detrimental impact of noise in gene expression. The reason for this is that the activity of the transcription factor is determined mainly by the relative concentrations of the kinase and its substrate and to a lesser extent by the concentration of the transcription factor itself. As a consequence, even relative small, uncorrelated fluctuations in these expression rates during the 2 hours prior to septation will result either in pre-septational activation of the transcription factor or failure to activate. Small fluctuations in the phosphatase expression rate on the other hand can be tolerated.

## 5. Outlook

(Molecular) biology has been incredibly successful in uncovering the regulatory principles and foundations of life while remaining largely a descriptive science. The key cellular machineries as well as the principles of cellular regulation have been revealed. Entire genomes have been sequenced, and the proteomes of important model organisms are currently being determined and quantified. Many interaction partners have been characterized [75, 76], and this has led to detailed wiring diagrams that describe the regulatory interactions in many important signalling pathways. The generation of large amount of data has necessitated the development of powerful bioinformatic tools to organize, analyse, store, and disseminate the available information and computational approaches are well established in these biological disciplines. In spite of huge amounts of data and powerful computational algorithms, it has remained difficult to predict biological functionalities and dependencies from the available data.

In particular, what we fail to understand is how sensitive the cellular responses are to variations in the signal and in the cellular proteome, how information is integrated by the cell, which set of downstream targets are activated (and which of these is important for the response of interest), and at what network components can sensitively control the output. This is the case, for instance, when a sensitive switch is observed and it remains unclear which combination of effects can yield the observed sensitivity in the system. The model for the *σ* ^*F*^ network could be applied to such a wide range of questions because the model had been parameterized so carefully based on detailed experimental information. While many signaling pathways have been modelled also in eukaryotes, including the MAPK, integrin, and TGF-beta signaling pathways [77–79] and much experimental information is available, realistic models of cellular signaling dynamics with predictive power as described in this paper are still largely missing.

One of the difficulties in generating predictive models is that most cellular information appears to be passed on without generating much “interesting” dynamics. Most cellular measurements reveal a slowly (or rapidly) increasing response of downstream signaling factors in response to a signal that may or may not fade after some time. A direct link, sometimes combined with a feedback can typically readily explain the data, and the experiments therefore contain insufficient information to uncover the intricate cross-talk between signaling components. Moreover, most models are typically based on experiments that have been conducted in different cell systems by different experimental groups. Since a single pathway typical can give rise to a wide range of different responses [80], it is difficult to obtain a consistent model from such varied datasets, and most studies thus again focus on the signaling capacity of signaling networks rather than on predictions of cellular behaviour under defined conditions. As a beneficial side effect of such efforts, important signaling paradigms, such as the importance of nuclear shuttling for the signaling response in eukaryotes [81, 82], have been uncovered.

Advances in microfluidics are likely to provide more and more consistent kinetic data to develop better models for cellular systems [83]. Eventually it will be important to link different detailed models and to integrate these into a multiscale model that puts the molecular regulation into the context of larger-scale processes such as tissue reorganization. Here a further important avenue will be the inclusion of spatial information to better understand the context in which cells signal. We have recently developed models for limb and lung development. In spite of largely missing kinetic information, mechanistic models with predictive power could be formulated [84, 85] because information on the spatiotemporal activation of gene expression in wildtype and mutant mouse embryos sufficiently constrained our models. A deeper understanding of biological mechanisms will require further careful modelling of well-characterized signaling networks in the functional context in which cells operate.
